# Preparation of Carbon-Based Solid Acid Catalyst from High-Sulfur Petroleum Coke with Nitric Acid and Ball Milling, and a Computational Evaluation of Inherent Sulfur Conversion Pathways

**DOI:** 10.3390/molecules28207051

**Published:** 2023-10-12

**Authors:** Qing Huang, Natalia M. Cabral, Xing Tong, Annelisa S. Schafranski, Pierre Kennepohl, Josephine M. Hill

**Affiliations:** 1 Department of Chemical and Petroleum Engineering, Schulich School of Engineering, University of Calgary, 2500 University Drive NW, Calgary, AB T2N 1N4, Canada; huangq.lpec@sinopec.com (Q.H.); natalia.marianocabra@ucalgary.ca (N.M.C.); annelisaschafranski@gmail.com (A.S.S.); 2 Department of Chemistry, University of British Columbia, Vancouver, BC V6T 1Z1, Canada; xing.tong@ucalgary.ca; 3 Department of Chemistry, University of Calgary, 2500 University Drive NW, Calgary, AB T2N 1N4, Canada; pierre.kennepohl@ucalgary.ca

**Keywords:** petroleum coke, sulfonic group, solid acid catalyst, DFT calculation, dibenzothiophene, nitric acid

## Abstract

A series of petroleum coke (petcoke)-derived solid acid catalysts were prepared via nitric acid treatment with or without ball milling pretreatment. The inherent sulfur in petcoke was converted to sulfonic groups, which were active sites for the esterification of octanoic acid and methanol at 60 °C, with ester yields of 14–43%. More specifically, samples without ball milling treated at 120 °C for 3 h had a total acidity of 4.67 mmol/g, which was 1.6 times that of the samples treated at 80 °C, despite their −SO_3_H acidities being similar (~0.08 mmol/g). The samples treated for 24 h had higher −SO_3_H (0.10 mmol/g) and total acidity (5.25 mmol/g) but not increased catalytic activity. Ball milling increased the defects and exposed aromatic hydrogen groups on petcoke, which facilitated further acid oxidation (0.12 mmol −SO_3_H/g for both materials and total acidity of 5.18 mmol/g and 5.01 mmol/g for BP-N-3/120 and BP-N-8/90, respectively) and an increased ester yield. DFT calculations were used to analyze the pathways of sulfonic acid group formation, and the reaction pathway with NO_2_• was the most thermodynamically and kinetically favourable. The activities of the prepared catalysts were related to the number of −SO_3_H acid sites, the total acidity, and the oxygen content, with the latter two factors having a negative impact.

## 1. Introduction

Petroleum coke (petcoke) is a carbon-rich (>80 wt%), low porosity by-product from oil sand upgrading processes. The impurities, particularly sulfur, in petcoke limit its use in industrial applications and as a fuel for combustion [1]. Stockpiling petcoke in the field is problematic because of the release of aromatic, volatile organic and other pollutant compounds to the atmosphere and/or groundwater [2,3]. In recent years, researchers have explored various applications for petcoke, including the co-gasification of petcoke with biomass or municipal solid waste [4,5,6], as an adsorbent after alkaline activation to increase its porosity [7,8,9,10,11], as a solid acid catalyst after sulfonation [12,13,14,15,16], and manufacturing carbon quantum dots [17]. We previously showed that the conversion of the inherent sulfur in petcoke to sulfonic acid groups enables catalytic activity for the esterification reaction of octanoic acid and methanol [16] but have not fully explored how this conversion may occur.

Esterification reactions are of current interest because they are used in the conversion of biomass, a renewable resource, to biodiesel, which is considered a cleaner alternative to fossil fuels. In general, this reaction generates esters and water via the reaction of free fatty acids and alcohols in the presence of a homogeneous or heterogeneous catalyst. Compared to conventional homogenous acid catalysts, solid acid catalysts can be separated and do not cause corrosion of the processing equipment. The preparation of solid acid catalysts from inexpensive and abundant carbon waste, including used tire rubber [18], agricultural waste [19,20,21,22], petcoke [12,13,14,15,16], and plastic waste [23], has been studied. The general preparation method involves sulfonation of the carbon materials with concentrated acids (e.g., H_2_SO_4_). The added −SO_3_H groups are the catalytically active sites for the reaction. Prior to sulfonation, most waste carbon materials were modified with carbonization, pyrolysis, or activation to increase the surface area and porosity [24]. These processes, however, generally remove some of the impurities, including sulfur, from petcoke [25]. An alternative method to modify the feeds but retain the sulfur is ball milling. This method was shown to expose aromatic hydrogen groups that promote the formation of −SO_3_H groups on the carbon [12].

In this study, the formation of the sulfonic groups on petcoke treated with nitric acid to convert the inherent sulfur was investigated. Several solid acid catalysts were prepared by varying different parameters—time, temperature, and pretreatment—characterized, and then their catalytic activity was determined for the esterification reaction between octanoic acid and methanol. To further understand the formation of the catalytically active sites, the sulfur conversion pathways were explored via density functional theory (DFT) calculations.

## 2. Results and Discussion

### 2.1. Impact of Treatment Time and Temperature

**Figure 1:**
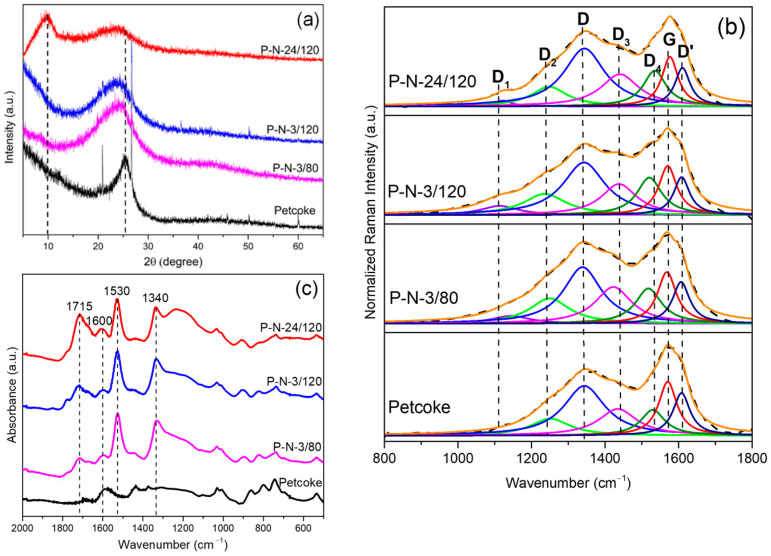
Physical and chemical characterization of petcoke and nitric acid treated petcoke samples. (**a**) XRD patterns; (**b**) Raman spectra; (**c**) FTIR spectra.

The structural features of the samples before and after functionalization were investigated by XRD and Raman spectroscopy. The XRD profiles (Figure 1a) illustrate the degree of oxidation of petcoke. A broad peak at ~25.5° 2θ corresponds to graphite (002). This peak broadened after nitric acid treatment, and the broadening increased with treatment time, with a left shift of the peak. According to Bragg’s law and the Scherrer equation, these changes are consistent with the expansion of interlayer spacing ($d_{002}$), possibly from intercalation of nitric acid and/or the formation of functional groups, and a decline in the crystallite stacking height (*Lc*) from exfoliation. Table 1 summarizes the $d_{002}$ and *Lc* values of the samples. The interlayer spacing of petcoke was 0.352 nm and increased to 0.364 nm, 0.376 nm, and 0.388 nm for samples P-N-3/80, P-N-3/120, and P-N-24/120, respectively. In parallel, the crystallite stacking height decreased from 2.14 nm for petcoke to 1.13 nm, and 1.06 nm and 0.95 nm for the functionalized samples. Increased treatment temperature and time resulted in more oxidation and exfoliation. A peak at ~10° 2θ on the profile of sample P-N-24/120 refers to graphene oxide structure and is consistent with added oxygen functionalities [26]. Since the oxidation by nitric acid disrupted the structure, the embedded impurities and microcrystals were removed, resulting in the disappearance of the sharpest peaks in the profiles of the acid-treated samples.

**Figure 2:**
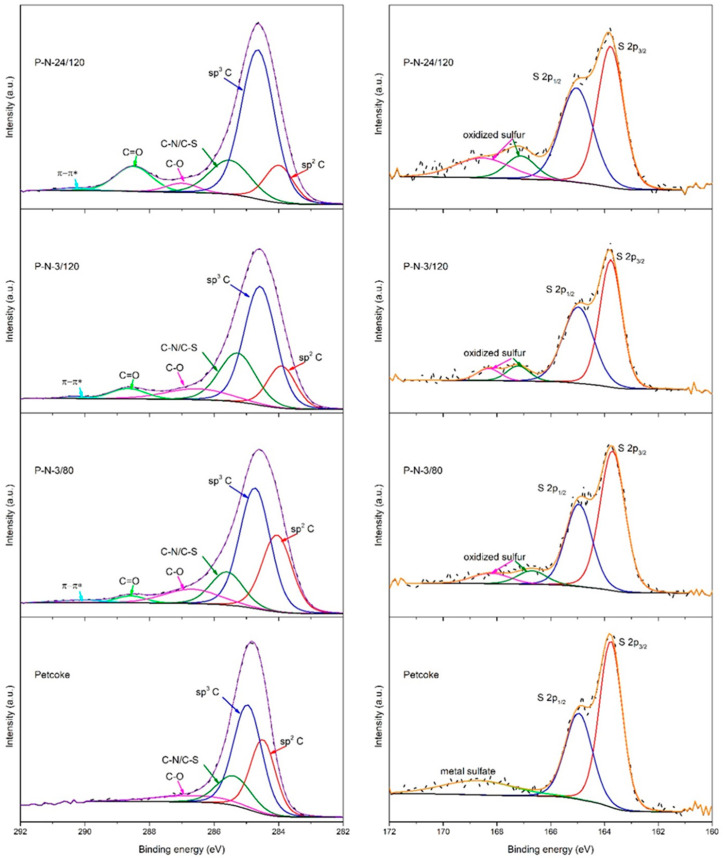
Deconvoluted high-resolution XPS spectra for petcoke and nitric acid modified samples: C1s (**left**) and S2p (**right**).

The Raman spectra (Figure 1b) contain broad peaks. The peaks between 1000–1800 cm^−1^ can be deconvoluted generally into six peaks with a Lorentzian function according to the investigation of Sadezky et al. [27]. The major peaks are centred at ~1340 cm^−1^ and 1575 cm^−1,^ assigned as D-band and G-band, respectively. The D-band represents the disordered graphitic structure (A_1g_-symmetry), whereas the G-band represents the ideal graphitic structure (E_2g_-symmetry). The I_D_/I_G_ ratio of petcoke was 0.92 and increased to 1.17 as the treatment time and temperature increased to 24 h and 120 °C, respectively, which was consistent with a disruption in the petcoke structure, as seen in the XRD profiles (Figure 1a). In addition to the main peaks, other peaks were located at ~1240 cm^−1^ (D_2_), ~1440 cm^−1^ (D_3_), ~1535 cm^−1^ (D_4_), and ~1610 cm^−1^ (D’); these deconvoluted peaks were considered evidence of the functional groups and hybridization in the graphene lattice as identified on other carbon materials, including graphene nanoribbons, graphene oxide-derived carbon quantum dots, functionalized graphene, and carbon nanotubes [28,29,30]. The intensities of the bands relative to the G band (i.e., I_D2_/I_G_, I_D3_/I_G_, I_D4_/I_G_*,* and I_D’_/I_G_) are shown in Table 1. The D’ band at ~1610 cm^−1^ is assigned to a graphitic lattice defect mode with E_2g_ symmetry [27], while the D_2_ band at ~1240 cm^−1^ is assigned to C-H bonds or C-OH functional groups. The I_D2_/I_G_ ratio generally increased after acid treatment but slightly decreased with the increase in treatment time, which may imply that the C-OH groups were further oxidized to COOH or C=O groups [29]. The D_3_ band and D_4_ band located between the D and the G bands are associated with the presence of oxygen and nitrogen groups [31]. The increase in both I_D3_/I_G_ and I_D4_/I_G_ ratios after acid treatment reflected the formation of these groups (consistent with the XPS results in Figure 2).

The crystallite size, *La*, decreased with increasing treatment time and temperature from 5.39 nm for petcoke to 4.24 nm for sample P-N-24/120 (Table 1). Petcoke samples treated for 1 h, 6 h, and 12 h at 120 °C were also analyzed. The relative area of the G band for all samples treated at 120 °C decreased up to a treatment time of 6 h and then remained relatively constant (Figure S1 in Supplementary Materials). The peak at ~1100 cm^−1^ after acid treatment (Figure 1b) is related to the sp^2^-sp^3^ bonds consistent with oxygen atoms covalently connecting at the sp^3^ carbon position from acid oxidation [27,29]. That is, the acid treatment enhanced the cleavage of the graphitic structures in petcoke and enhanced the formation of sp^2^-sp^3^ bonds. P-N-3/80 had a smaller I_D_/I_G_ ratio and larger *La* value than those of P-N-3/120, consistent with the higher temperature treatment increasing the chemical cleavage of the petcoke structure.

Petcoke contains ultra-micropores, so the surface area and pore volume were measured via CO_2_ adsorption; the results are given in Table 1. Overall, acid treatment led to an increase in surface area and pore volume. The I_D_/I_G_ ratio of sample P-N-3/80 was 0.97 and only slightly increased from the value of 0.92 for petcoke, but the surface area and pore volume of sample P-N-3/80 were 231 m^2^/g and 0.063 cm^3^/g, respectively, which were approximately three times the corresponding values for petcoke. Compared with petcoke, the significant increase for acid-treated samples in surface area and porosity, therefore, was not due to the change in the carbon structure but was likely a result of the removal of mineral impurities. The TGA analysis confirmed a decrease in ash content after acid treatment (Figure S2). Longer treatment times and a higher treatment temperature, however, reduced the porosity. Specifically, the surface area and pore volume decreased from 217 m^2^/g and 0.061 cm^3^/g for sample P-N-3/120 to 151 m^2^/g and 0.045 cm^3^/g for sample P-N-24/120. The increased degree of oxidation resulted in structural collapse and the generation of oxygen-containing groups blocking pores and inhibiting adsorption (Figure S3) [32,33,34].

The chemical properties were analyzed via FTIR and XPS. Most peaks in the FTIR spectra were in the range of 2000–600 cm^−1^. In Figure 1c, peaks between 600–900 cm^−1^ corresponded to aromatic hydrogen, and the peak intensity in this range decreased after acid treatment (Table 2) because of electrophilic substitution by HNO_3_ and the oxidation of petcoke. Peaks centred at 1600 cm^−1^ correspond to poly-aromatic rings of hydrocarbon molecules in petcoke, while peaks at ~1030 cm^−1^ and ~1200 cm^−1^ correspond to the S=O stretching vibrations of sulfonic groups [35]. The spectra of the acid-treated samples contained peaks at 1340 cm^−1^ and 1530 cm^−1^ corresponding to *ν_s_* (NO_2_) and *ν_as_* (NO_2_), respectively [36], and at 1715 cm^−1^ corresponding to C=O stretching in carboxylic groups. The latter band was the most intense in the spectrum of sample P-N-24/120. The main surface groups on the acid-treated petcoke, therefore, are sulfonic groups, nitro groups and carboxylic groups.

The surface elemental composition was determined using an XPS survey scan (Table 2), and the predominant elements were C and O, but N and S were also present. Petcoke also contained Si (2.7 at%) and Al (1.5 at%), but these elements were not evident after acid treatment, consistent with the XRD (Figure 1a) and TGA analyses (Figure S2). As expected, the nitrogen content increased after treatment with HNO_3_ while the sulfur content decreased, likely due to oxidative desulfurization since HNO_3_ is a strong oxidant. The O/C ratio increased significantly more as the treatment time or temperature increased compared to the decrease in the S/C ratio.

The oxidation states of the carbon and sulfur in the samples were determined via deconvoluting XPS high-resolution spectra (Figure 2) [37]. The C 1s high-resolution spectra (Figure 2 left) contain the most intense peaks centred at ~284.7 eV for the sp^3^ carbon and at ~284.1 eV for the sp^2^ carbon. The ratio of C_sp_^3^/C_sp_^2^ increased from 1.57 for petcoke to 1.6, 3.2 and 4.6 for samples P-N-3/80, P-N-3/120 and P-N-24/120, respectively. The peak at ~285.5 eV corresponds to carbon hybridized with S and N. The spectra also contain two relatively low-intensity peaks associated with C-O and C=O at ~286.6 eV and ~288.4 eV, respectively. The percentage of carbon in C-O bonds on petcoke was 8.9%, with no carbon in C=O bonds. Thus, the surface groups on petcoke include hydroxyl, ether, and epoxy groups. The percentage of carbon in C-O bonds increased to 9.7% with up to 3 h of acid treatment but decreased to 3.4% after 24 h of treatment. C=O bonds were contained in the spectra of the acid-treated samples and increased with treatment time, from 4.6% after 3 h to 10.5% after 24 h. In addition, temperature affected the existence of oxygen-containing functional groups. Compared with the samples treated at 120 °C, the samples treated at 80 °C have more C-O bonds (9.7%) and fewer C=O bonds (2.5%). A broad peak at ~290 eV associated with π-π* transition was evident for the acid-treated samples. Regarding the sulfur content, the high-resolution S2p peaks of all samples (Figure 2 right) have peaks at binding energies of ~163.8 eV and ~165 eV that are characteristic of S 2p_3/2_ and 2p_1/2_ of thiophene-S [38]. The acid-treated samples have peaks at ~167 eV and 168.4 eV related to oxidized sulfur [39], where the latter is assigned to −SO_3_H, confirming that during the functionalization, the inherent sulfur in petcoke can be converted to sulfonic acid.

### 2.2. Effect of Ball Milling Pretreatment

**Figure 3:**
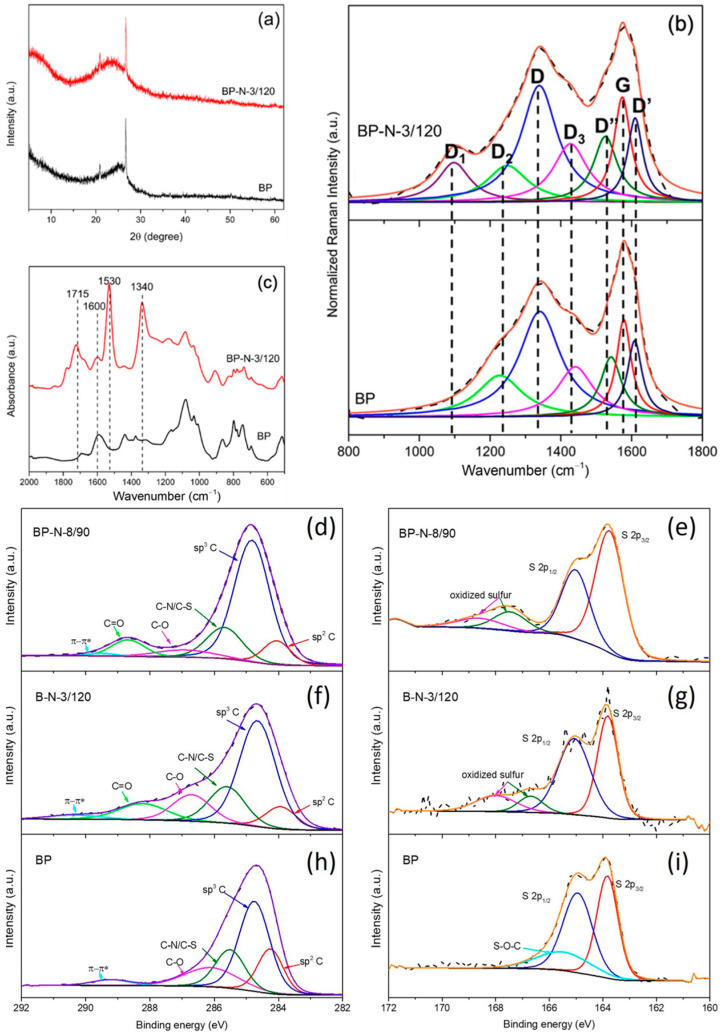
Physical and chemical characterization of ball-milled petcoke (BP) and nitric acid modified ball-milled petcoke (BP-N-3/120 and BP-N-8/90): (**a**) XRD patterns; (**b**) FTIR spectra; (**c**) Raman spectra; and (**d**–**i**) deconvoluted high-resolution XPS spectra of C 1s (**d**,**f**,**h**) and S 2p (**e**,**g**,**i**).

Ball milling pretreatment resulted in both structural and chemical changes to petcoke (Figure 3, Table 1 and Table 2). Ball milling did not change the interlayer spacing ($d_{002}$) but did promote the exfoliation of petcoke. That is, after ball milling, *Lc* decreased to 1.47 nm (Table 1). Ball milling also resulted in a broadening of the XRD band at ~25.5° 2θ for petcoke with further broadening after acid treatment at 120 °C and 3 h (Figure 3a), as seen for the samples without ball milling (Figure 1a). There was a corresponding decrease in *Lc* to 0.97 nm, which was comparable to the *Lc* value of sample P-N-24/120. Ball milling also increased the number of defects (i.e., increased I_D2_/I_G_, I_D3_/I_G_, I_D4_/I_G_ and I_D’_/I_G_ ratios, Table 1). The significant decrease in *La* from 5.39 nm to 4.59 nm, which is even smaller than that for sample P-N-3/120, reflected the lattice cleavage introduced by ball milling. The value of *La* decreased to 4.47 nm for sample BP-N-3/120, and there was a significant increase in the D_1_ band intensity (Figure 3b). The surface area and pore volume increased after ball milling to 172 m^2^/g and 0.056 cm^3^/g, respectively.

In addition to the structural changes, ball milling impacted the surface chemical composition via the exposure of more aromatic H and the addition of oxygen on the surface of the petcoke, which facilitated the subsequent reaction with acid. That is, samples BP-N-3/120 and BP-N-8/90 had higher O and N content, −SO_3_H acidity and total acidity than the other samples (Table 2). In Figure 3c, the appearance of peaks between 1000–1200 cm^−1^ for the ball-milled petcoke is consistent with the increased oxygen content. Ball milling introduced π-π* transition to petcoke (Figure 3h). Although petcoke was oxidized via ball milling, −SO_3_H species were not generated. Instead, a new S peak at ~165.5 eV, ascribed to a S-O-C bond, was identified on the ball-milled petcoke (Figure 3i).

### 2.3. Esterification Reaction

**Figure 4:**
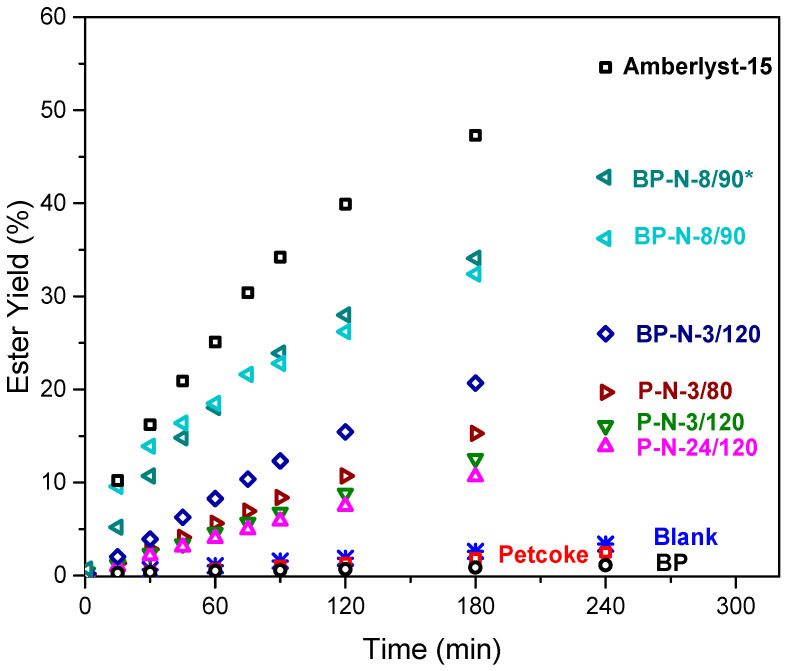
Esterification of octanoic acid using petcoke-derived catalysts. Reaction conditions: methanol: octanoic acid = 20:1 M ratio, 60 °C, 800 rpm, 5 wt% of catalyst. * methanol: octanoic acid = 10:1 M ratio.

All samples were tested in the esterification reaction between octanoic acid and methanol, and the results are shown in Figure 4. The 4 h yield of methyl octanoate decreased from 3.5% for the blank test to 2.5% with petcoke and 1.1% with ball-milled petcoke. The hydrogen bonding acceptors in esters can bond with oxygen-containing groups on carbon materials, resulting in the adsorption of the reaction product and explaining these results [40,41]. After acid treatment, the activity improved in the order of samples P-N-24/120 < P-N-3/120 < P-N-3/80 < BP-N-3/120. As the yields on these catalysts were less than half of that on a commercial Amberlyst-15 catalyst, the preparation parameters were varied further to produce catalyst BP-N-8/90 (i.e., 8 h treatment with nitric acid at 90 °C). The ester yield on this catalyst increased to ~36% after 4 h. Decreasing the methanol: octanoic acid molar ratio from 20:1 to 10:1 further increased the yield to ~43%; this higher conversion can be explained by a better dispersion of the catalyst in this condition. The TOF were between 64–143 h^−1^, generally consistent with more −SO_3_H groups improving the reactivity. When samples possess a similar level of total acidity, the sample with more −SO_3_H groups had a higher TOF, such as BP-N-3/120 and BP-N-8/90, compared to P-N-24/120. More specifically TOF of 114 h^−1^, 105 h^−1^, and 64 h^−1^ were obtained with samples P-N-3/80, P-N-3/120 and P-N-24/120, respectively. The TOF is negatively correlated with total acidity, suggesting that adsorption of the reaction products—water and methyl octanoate—by oxygen-containing functional groups on the samples hinders further reaction. Although the oxygen content and total acidity of the sample, BP-N-3/120 were higher than those of samples P-N-3/80 and P-N-3/120, its TOF was 114 h^−1^, which was higher than that of P-N-3/120 and equal to that of P-N-3/80. This result was likely because the ball-milled samples had more −SO_3_H groups, and when comparing the TOF of these materials, which have the same sulfonic acid sites concentration (0.12 mmol/g), the BP-N-8/90 has a higher TOF of 143 h^−1^ due to its lower total acidity of 5.01 mmol/g against 5.18 mmol/g for BP-N-3/120.

Therefore, for petcoke-derived solid acid catalysts, it is necessary to increase the number of −SO_3_H groups while reducing other oxygen-containing acid functional groups. The results from other recent studies are summarized in Table S1. Carbon-based solid acid catalysts have productivities varying between ~1 and 10 g_product_·g_catalyst_^−1^·h^−1^, which are much lower than that achieved with sulfuric acid, 52 g_product_·g_catalyst_^−1^·h^−1^. In terms of the estimated TOF, the BP-N-8/90 catalyst has a higher value of 143 h^−1^ than the other materials (Table S1). Although some catalysts have a higher conversion—sulfonated chitosan (83%, [42]), hollow sulfonated mesoporous carbon spheres (70%, [43]), and sulfonated spent coffee grounds (~90% [44])—the catalysts were tested at less desirable conditions that include using a higher alcohol excess, which increases the ester production cost [42,43], and higher reaction temperatures [43,44], and/or their preparation method was more complex for productivity similar or lower than that achieved by the petcoke-derived catalyst. From a molecular point of view (TOF), the sites (−SO_3_H) generated from the inherent sulfur in petcoke are more active than those on Amberlyst-15, and the activity, although lower, is comparable to that generated from the sulfonation process. However, the productivity of these petcoke catalysts without additional −SO_3_H sites is lower than Amberlyst-15 and the catalysts prepared from sulfonation, suggesting that it is critical to increase the number of −SO_3_H groups. A better understanding of the mechanism of sulfur conversion in petcoke is required to improve the treatment strategy and increase the number of −SO_3_H groups. DFT calculations were carried out to investigate the possible sulfur conversion pathways.

### 2.4. Exploration of Sulfonic Group Formation Mechanisms

**Figure 5:**
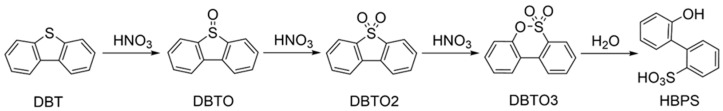
Possible general reaction pathway for the oxidation of DBT.

The formation of −SO_3_H was verified using XPS and esterification reactions. The sulfur in petcoke mainly exists in the form of thiophene, so dibenzothiophene (DBT) was selected as a model compound in this study. The possible reaction pathway for the oxidation of DBT (Figure 5) builds on a study by D’Alessandro et al. [45]. DBT is oxidized to dibenzothiophene oxide (DBTO) and dibenzothiophene dioxide (DBTO_2_), followed by further oxidation to biphenylsultone (DBTO_3_), which is then hydrolyzed to 2-(20-hydroxybiphenyl)sulfonate (HBPS). In particular, the conversion from sulfone to sultone was proposed as an analogous Baeyer-Villiger (B–V) oxidation [45,46], during which the electron-deficient sulfur atom was further attacked by oxidizers to form the sultone structure.

Based on the structure of DBT, we envisaged two possible pathways for sulfur activation: (i) sulfur acting as a donor via its valence lone pairs and (ii) sulfur acting as an acceptor via chalcogen bonding [47,48]. The latter situation results from the presence of a “σ-hole” (area of positive electrostatic potential) at the extension of each C-S bond. These electrophilic σ-holes are potential sites for nucleophilic attack. The ability of DBT to act as a donor and/or an acceptor depends strongly on the nature of the species with which it reacts. Given the dissociation of HNO_3_ in aqueous environments [49], both HNO_3_ and NO_3_^−^ were considered potential reagents in the reaction with DBT.

**Figure 6:**
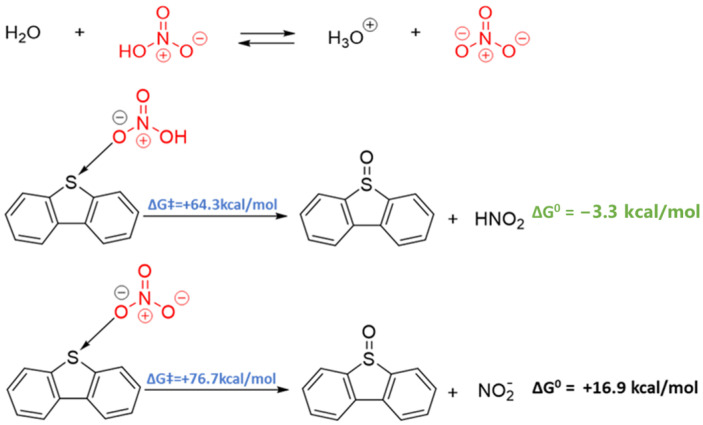
Reaction profile for the comparison of DBT reacted with nitric acid and nitrate anion.

In both cases, the interaction between the nitrogen and sulfur-containing species was initiated via chalcogen bonding and allowed for a single-step oxygen transfer reaction to form DBTO with high activation barriers (see Figure 6). Both kinetically and thermodynamically, however, this first oxidation is preferable from the acid form, which suggests that measures to avoid hydrolysis (e.g., via the addition of H_2_SO_4_) should lead to better conversion if oxidation occurs in this manner. Further oxidation with HNO_3_ and/or NO_3_^−^ was also explored, and details of the calculated reaction profiles are shown in Figure S4 (see Supplementary Materials). The formation of DBTO_2_ is more facile than the first oxidation, but the formation of DBTO_3_ proceeds via a very high energy transition state that is almost 100 kcal/mol (418.6 kJ/mol) higher in energy than the DBT starting material. In order for this reaction to proceed, catalysis would be required to allow for conversion from DBT → DBTO_3_. Notably, iron is present in trace amounts in petcoke [1] and was shown to be an effective catalyst for DBT oxidation [50,51].

From the results of the XPS analysis, the nitrate groups were detected on the acid-treated samples, supporting that nitration reactions occurred during the acid treatment (Figure S5). On the basis of the nitration mechanism, the nitration agents nitronium ion (NO_2_^+^) and nitrogen dioxide radical (NO_2_•) were generated from HNO_3_.

The formation of a brown gas, consistent with the formation of nitrogen dioxide (NO_2_), is observed during acid treatment. The potential role of either NO_2_• or NO_2_^+^ in DBT oxidation, therefore, was explored. As with HNO_3_ and NO_3_^−^, the interaction of these species with DBT proceeds via chalcogen bonding that places the reactive oxygen species with the sulfur atom (see Figure S6). The reaction with NO_2_^+^ to form DBTO and DBTO_2_ is significantly more facile than that observed from HNO_3_/NO_3_^−^ but any path to the formation of DBTO_3_ could not be found, presumably because of the increasing steric hindrance around the sulfur atom and a concomitant large increase in Pauli repulsion between the reagents.

**Figure 7:**
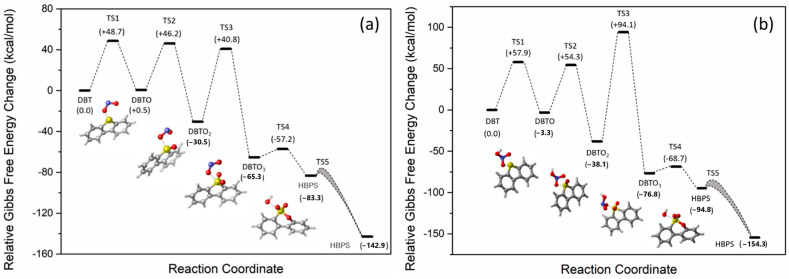
Energy diagram of DBT oxidized by (**a**) NO_2_• and (**b**) HNO_3_ at 25 °C. Sulfur, Nitrogen, Oxygen, Hydrogen and Carbon are represented by yellow, blue, red, white and gray atoms respectively.

The reaction with NO_2_, however, is both thermodynamically and chemically quite reasonable (see Figure 7b). The three oxidation processes can all proceed with transition states that are <50 kcal/mol above that of the starting materials (DBT + NO_2_). Of all the pathways evaluated, this is the kinetically most accessible pathway. The formation of HO• was also reported to be generated during the process of HNO_3_ decomposition into nitrogen oxides [52]. The radical HO• is highly reactive with strong oxidizing properties, which can not only react with sulfur in petcoke but also with carbon, even graphitic carbon. Thus, HO• might also contribute to the overall oxidation process.

Following the formation of DBTO_3_, with the presence of H_2_O or OH^−^ the ring structure of sultone can be opened. The sulfonic groups and hydroxyl groups will be formed immediately in an acid environment, associated with a significant downhill process (−77.5 kcal/mol). In the presence of water, however, a spontaneous desulfurization reaction with ∆G^0^ of −0.4 kcal/mol and a marginal energy barrier led to the removal of the sulfonic group (Figure S7). Therefore, SO_2_ will be generated in the washing step via the reaction of −SO_3_H groups with OH^−^. To mitigate the production of SO_2_, a slightly acidic environment with a shorter contact time can be used for washing. In addition, H_2_O is a product of the esterification reaction and may be responsible for the loss of the active sites (−SO_3_H groups) during the reaction. In order to retain catalytically active sites, improve the stability of the catalysts, and prevent the production of SO_2_, the produced water must be removed by modifying the reaction system. Molecular sieves were proposed to adsorb the water in systems using ethanol [53] but resulted in decreased conversion with methanol in our system.

Although using nitric acid to modify petcoke will generate some air pollutants such as SO_2_ and NO_2_, it is an active oxidant that converts petcoke from solid waste to a catalyst using the inherent sulfur to produce −SO_3_H groups. Since HO• can contribute to the oxidation process, H_2_O_2_ could be a more environmentally friendly alternative. The intercalation effect of nitric acid, however, enlarges the interlayer spacing, promoting access to more sulfur. Thus, the proposed method can be considered an efficient method of waste-to-catalyst conversion, but further optimization and improvements in the process are required. In particular, the preparation conditions must be adjusted to favour the desired reaction by adding a catalyst that promotes the formation of NO_2_• or selecting another oxidant that can generate radicals which have similar properties as NO_2_•.

## 3. Materials and Methods

### 3.1. Materials

Delayed petcoke (Suncor Energy Inc., Calgary, AB, Canada) was ground and sieved to particle sizes below 150 μm. Nitric acid (HNO_3_, 68–70%) was purchased from VWR International. Hydrochloric acid (HCl, 37%) and sodium hydroxide (NaOH, 0.1 M solution in water) were purchased from Sigma-Aldrich (Markham, ON, Canada) and used for the titration of the acid sites.

### 3.2. Preparation of Acid-Functionalized Samples

Samples were prepared as described previously [16]. Briefly, 2 g petcoke was treated with 20 mL HNO_3_ for 3 h or 24 h at 120 °C. The mixtures of acid and petcoke were filtered using a 0.22 µm membrane filter, and the obtained solid substrates were washed with reverse osmosis (RO) water until neutral pH was achieved, followed by drying at 120 °C overnight. The samples were named according to this treatment. For example, sample P-N-24/120 refers to petcoke (P) treated with nitric (N) acid for 24 h at 120 °C. An additional sample was prepared for 3 h at 80 °C and named P-N-3/80. To investigate the impact of ball milling before the acid treatment, a sample of petcoke was ball milled with 5 mm zirconia balls at a sample-to-ball mass ratio of 1:25 at 300 rpm for 4 h and named BP. A portion of this ball-milled petcoke was treated with HNO_3_ for 3 h at 120 °C or 8 h at 90 °C, and these samples were named BP-N-3/120 and BP-N-8/90. In all cases, the yield (by mass of catalyst) was over 90%.

### 3.3. Characterization

The structural features of petcoke-derived samples were investigated by X-ray diffraction (XRD, Rigaku Miniflex II benchtop PXRD, Rigaku Corporation, Tokyo, Japan) using a CuKα X-ray source (λ = 0.15405 nm). The sample powder was dispersed with isopropanol, and a drop was placed onto the sample holder. In addition to XRD, samples were analyzed with a WiTec Alpha-300 series confocal Raman microscope (Knoxville, TN, USA) with a 532 nm diode laser. The interlayer distances (*d*) and crystallite sizes (*La*) were determined with standard methods [37,54,55,56].

Sample porosities were measured via CO_2_ adsorption at 0 °C using a Tristar 3000 instrument (Micromeritics Instrument Corporation, Norcross, GA, USA). The Solution of the Adsorption Integral Equation Using Splines (SAIES) software version 2.02 was used to calculate the surface area and pore volume. The 2D Non-Local Density Functional Theory with heterogeneous surfaces model (2D-NLDFT-HS) provided the best fit for the adsorption data. All samples were degassed under vacuum at 150 °C prior to adsorption analysis.

The ash content and thermostability of petcoke-derived samples were analyzed via thermogravimetric analysis (TGA, SDT Q600, TA Instruments, New Castle, DE, USA). Approximately 0.015 g of the dried sample was heated from ambient temperature to 750 °C at 20 °C/min in a 50 mL/min flow of air and held at 750 °C for 0.5 h.

A Fourier Transform Infrared (FTIR, Nicolet iS50, Thermo Fisher Scientific, Waltham, MA, USA) spectrometer with an attenuated transmission reflectance (ATR) was used to determine the surface groups on the samples before and after treatment. The ATR spectra were collected in the 4000 to 400 cm^−1^ wavenumber range, accumulating 32 scans at 4 cm^−1^ resolution. Aromatic hydrogen content was estimated by the ATR spectra method established by Ye et al. [13]. The peaks at 600–900 cm^−1^ are affected by the aromatic hydrogen content of the samples. Dibenzothiophene (DBT) was used as a model compound to establish a correlation between spectral area and the aromatic hydrogen content in petcoke. Further surface analysis was conducted with X-ray photoelectron spectroscopy (XPS, Kratos Axis spectrometer, Kratos Analytical Limited, Manchester, UK) with monochromatized Al Kα (hυ = 1486.71 eV) radiation. Compositions were calculated from the survey spectra using the major elemental peaks. The software CasaXPS (Version 2.3.22PR1.0, Casa Software Ltd., Teignmouth, UK) was used for component analysis to deconvolute the C1s and S2p peaks. The number of total surface acidic groups on the samples was determined by a modified Boehm titration that involved adding 0.1 g of sample to 5 mL of a 0.1 M NaOH solution. The mixture was oscillated in a shaker (VWR Symphony 5000I Shaker, Henry Troemner LLC, Thorofare, NJ, USA) at 25 °C and 250 rpm for 24 h. This solution was then titrated with 0.01 M HCl solution using phenolphthalein as the indicator.

### 3.4. Esterification Reaction

The reaction between octanoic acid and methanol, Equation (1), was used to test catalyst activity. Reactions were carried out in a two-neck round bottom flask (100 mL) housed in a water bath, stirred magnetically, and jacketed by a condenser. First, 0.2 g of catalyst, 5 mL of octanoic acid, and 1 mL of dodecane (internal standard) were added to the reaction flask and heated to 60 °C while stirring at 800 rpm for 4 h. The amount of catalyst used corresponds to approximately 5 wt% of the system (i.e., ~0.2 g catalyst and 4.4 g octanoic acid). At time zero, 25 mL of methanol was added to the reaction mixture via an adapter on the side neck. Periodically, ~0.4 mL samples of the suspension were withdrawn by syringe (the total sampling decreased the initial reaction volume by less than 15%). The catalyst was separated by passing the suspension through a membrane filter (0.22 µm, EZFlow^®^, Foxx Life Sciences, Londonderry, NH, USA) to prevent further reaction. Next, 0.1 µL of solution was injected into a gas chromatography with a flame ionization detector (GC-FID, 6890N, Agilent Technologies, Santa Clara, CA, USA) equipped with a DB FATWAX UI capillary column (polyethylene glycol-type, 30 m × 0.25 mm × 0.25 µm). The temperature program for the GC analysis consisted of 0.5 min at 50 °C, heating at 40 °C/min to 200 °C and holding for 2 min at 200 °C. Helium was used as the carrier gas (injection using split mode with a ratio of 50:1). The temperatures of the injector and detector were set at 250 and 300 °C, respectively. The detector mode used was constant makeup flow, and the error was estimated to be <1% for all samples as determined by triplicate analysis of several aliquots.

(1) $$C_{7}H_{15}COOH+{CH}_{3}OH↔C_{7}H_{15}COOCH_{3}+H_{2}O$$

The ester yield was calculated based on the following equation:

(2) $$\%yield=\frac{m_{actual}}{m_{theoretical}}\times 100\%$$

where $m_{actual}$ is the mass of caprylic acid methyl ester formed and $m_{theoretical}$ is the theoretical maximum mass of ester. The initial turnover frequencies (*TOF*) were calculated as follows [57]:

(3) $$TOF=\frac{k\times {\left[OA\right]}_{0}\times V}{m_{catalyst}\times \left[-{SO}_{3}H\right]}$$

where k is the pseudo first-order rate constant (min^−1^), ${\left[OA\right]}_{0}$ is the initial concentration of octanoic acid (mmol/mL), V is the volume of the reaction solution (mL), $m_{catalyst}$ is the mass of catalyst added (g) into the reactor, and [−SO_3_H] is the concentration of sulfonic groups on the catalyst surface (mmol/g). The pseudo first-order model (Equation (4)) assumes that the reverse rate constant is negligible [58] and the methanol concentration is constant [13,58]:

(4) $$\left[OA\right]={\left[OA\right]}_{0}e^{-kt}$$

where [*OA*] is the concentration (mmol/mL) of octanoic acid at time *t* (min) and *k* is the pseudo first-order rate constant (min^−1^). The model assumptions are satisfied at the initial stages of the reaction and with the conditions used.

### 3.5. DFT Calculations

Dibenzothiophene (DBT) was chosen as a model compound, and DFT calculations were run using the Gaussian 09 (G09) computational package [59]. All geometry optimizations were performed in G09 using the restricted M06-2X functional and the Def2-TZVP basis set [60,61]. In radical species, the restricted open-shell formalism was employed using the same functional and basis sets as for other species [62]. A conductor-like polarizable continuum solvation model (CPCM) of methanol and an ultrafine integration grid were applied. Geometry optimizations were performed on all species, and frequency calculations were performed at the same level of theory to confirm the absence of imaginary frequencies. Transition states were identified and optimized using the quadratic synchronous transit (QST) approach [63]. All structures labelled as transition states exhibit one imaginary frequency.

## 4. Conclusions

This study explored the details of using the inherent sulfur in petcoke to prepare solid acid catalysts for the esterification reaction between octanoic acid and methanol by oxidizing petcoke to obtain the catalytic active site—sulfonic groups. According to XPS analysis and the yield of methyl octanoate, some sulfur was converted into −SO_3_H groups by nitric acid treatment. While not a green solvent, nitric acid was effective in exfoliating the petcoke and making the sulfur accessible. The effect of treatment time and temperature was investigated, as well as the effect of ball milling pretreatment. Either a longer time or a higher temperature treatment led to an increase in oxygen-containing groups that contributed to the total acidity, while only the longer treatment time had an effect on the −SO_3_H acidity. Comparing 80 °C and 120 °C, the resulting −SO_3_H acidity was similar: ~0.08 mmol/g for the catalysts without ball-milling treatment. From the results of the esterification reaction, however, the yield of the product is not only related to the number of sulfonic groups but shows a negative correlation with oxygen-containing functional groups, implying that the adsorption effect of oxygen-containing functional groups might hinder the esterification reaction. Pretreatment with ball milling exposed defects and increased the amount of exposed aromatic hydrogen, which promoted the subsequent acid treatment to obtain more sulfonic acid groups and a more active catalyst; BP-N-8/90 for instance, resulted in an ester yield of 43% after 4 h. This yield was less than that obtained with an Amberlyst-15 catalyst (~55%), but the petcoke-derived catalyst was prepared from waste material and is not yet optimized. The DFT calculation results indicated that NO_2_^+^ and NO_2_• not only played a critical role in nitration during acid treatment but also contributed to the oxidation of the inherent sulfur in petcoke.

## Figures and Tables

**Table 1:** Summary of structural properties determined by XRD and Raman for petcoke before and after functionalization with nitric acid.

	$d_{002}$ ^1^ (nm)	*Lc* ^2^ (nm)	*La* ^3^ (nm)	I_D_/I_G_	I_D2_/I_G_	I_D3_/I_G_	I_D4_/I_G_	I_D’_/I_G_	Surface Area ^4^ (m^2^/g)	Pore Volume ^4^ (cm^3^/g)
Petcoke	0.352	2.14	5.39	0.92	0.32	0.49	0.48	0.77	84	0.021
P-N-3/80	0.364	1.13	5.11	0.97	0.49	0.71	0.68	0.81	231	0.063
P-N-3/120	0.376	1.06	4.64	1.07	0.43	0.63	0.76	0.77	217	0.061
P-N-24/120	0.388	0.95	4.24	1.17	0.39	0.65	0.72	0.78	151	0.045
BP	0.351	1.47	4.59	1.08	0.43	0.52	0.62	0.78	172	0.056
BP-N-3/120	0.379	0.97	4.47	1.11	0.35	0.56	0.63	0.80	200	0.054

^1^ Calculated from Bragg’s law; ^2^ Calculated from Scherrer equation; ^3^ Calculated from Tuinstra and Koening’s relationship; ^4^ Based CO_2_ adsorption.

**Table 2:** Surface chemical properties of petcoke before and after various acid treatments.

Sample	Elemental Analysis (at%) ^1^						Acidity (mmol/g)		Aromatic Hydrogen (mmol/g) ^3^	Esterification Results	
	C	O	N	S	O/C	S/C	−SO_3_H ^1^	Total ^2^		Ester Yield ^4^ (%)	TOF (h^−1^)
Petcoke	74.5	18.0	1.1	2.2	0.24	0.030	nd ^5^	0.34 (0.01)	1.5 (0.1)	2.5	- ^7^
P-N-3/80	73.6	19.4	5.3	1.7	0.26	0.023	0.08	3.60 (0.09)	1.1 (0.1)	19.7	114
P-N-3/120	72.5	21.4	4.4	1.7	0.30	0.023	0.07	4.67 (0.16)	1.1 (0.1)	16.1	105
P-N-24/120	68.5	26.2	3.9	1.4	0.38	0.020	0.10	5.25 (0.23)	0.9 (0.1)	13.9	64
BP	73.9	20.8	0.9	2.3	0.28	0.030	nd	0.55 (0.03)	2.1 (0.2)	1.1	- ^7^
BP-N-3/120	69.7	23.5	5.3	1.5	0.34	0.024	0.12	5.18 (0.28)	2.0 (0.2)	26.0	114
BP-N-8/90	64.2	29.1	5.4	1.3	0.45	0.020	0.12	5.01 (0.08)	1.7 (0.2)	36.2/42.8 ^6^	143

^1^ Estimated from XPS results; ^2^ Estimated by Boehm titration, the values in brackets are the standard deviations determined from three samples; ^3^ Estimated by FTIR analysis (ATR), the values in brackets are the standard deviations determined from three samples; ^4^ after 4 h; ^5^ nd: not detected; ^6^ methanol: octanoic acid molar ratio 10:1, ^7^ TOF could not be calculated for materials without −SO_3_H acidity.

## Data Availability

The data presented in this study are available on request from the corresponding author.

## Supplementary Materials

The following supporting information can be downloaded at https://www.mdpi.com/article/10.3390/molecules28207051/s1. Figure S1: Relative area of the G band in the Raman spectra of acid-treated percoke in percentage of total area in the 1000–1800 cm^−1^ region; Figure S2: TGA profiles of petcoke and acid-treated petcoke (20 °C/min to 750 °C in a 50 mL/min flow of air); Figure S3: Pore size distributions of petcoke and petcoke-derived samples, as determined by CO_2_ adsorption using a 2D-NLDFT-HS model; Figure S4: Reaction profile of DBT reacted with nitric acid (HNO_3_); Figure S5: Deconvoluted high-resolution XPS spectra of N 1s; Figure S6: Reaction profile of DBT reacted with nitronium ion (NO_2_^+^); Figure S7: A spontaneous desulfurization reaction; Table S1: Esterification of octanoic acid with methanol over different acid catalysts. References [64,65,66] are cited in the supplementary material.
