# Supplementary material for: Preparation of Carbon-Based Solid Acid Catalyst from High-Sulfur Petroleum Coke with Nitric Acid and Ball Milling, and a Computational Evaluation of Inherent Sulfur Conversion Pathways
Source: Molecules. 2023 Oct 12;28(20):7051. doi: 10.3390/molecules28207051 (PMC10609519; doi:10.3390/molecules28207051)
Supplement: Supplementary file 1 [file molecules-28-07051-s001.zip › molecules-2601832-supplementary.pdf]

Electronic Supplementary Material for

Preparation of Carbon-Based Solid Acid Catalyst from High-Sulfur Petroleum Coke with Nitric Acid and Ball Milling, and a Computational Evaluation of Inherent Sulfur Conversion Pathways

Qing Huang [1], Natalia M. Cabral [1], Xing Tong [2,3], Annelisa S. Schafranski [1], Pierre Kennepohl [3] and Josephine M. Hill [1,*]

[1] Department of Chemical and Petroleum Engineering, Schulich School of Engineering, University of Calgary, 2500 University Drive NW, Calgary, AB T2N 1N4, Canada; huangq.lpec@sinopec.com (Q.H.); natalia.marianocabra@ucalgary.ca (N.M.C.); annelisaschafranski@gmail.com (A.S.S.)

[2] Department of Chemistry, University of British Columbia, Vancouver, BC V6T 1Z1, Canada; xing.tong@ucalgary.ca

[3] Department of Chemistry, University of Calgary, 2500 University Drive NW, Calgary, AB T2N 1N4, Canada; pierre.kennepohl@ucalgary.ca

* Correspondence: jhill@ucalgary.ca; Tel.: +1-403-210-9488; Fax: +1-403-284-4852

Number of pages: 6

Number of figures: 7 (Figure S1-S7)

Number of tables: 1 (Table S1)

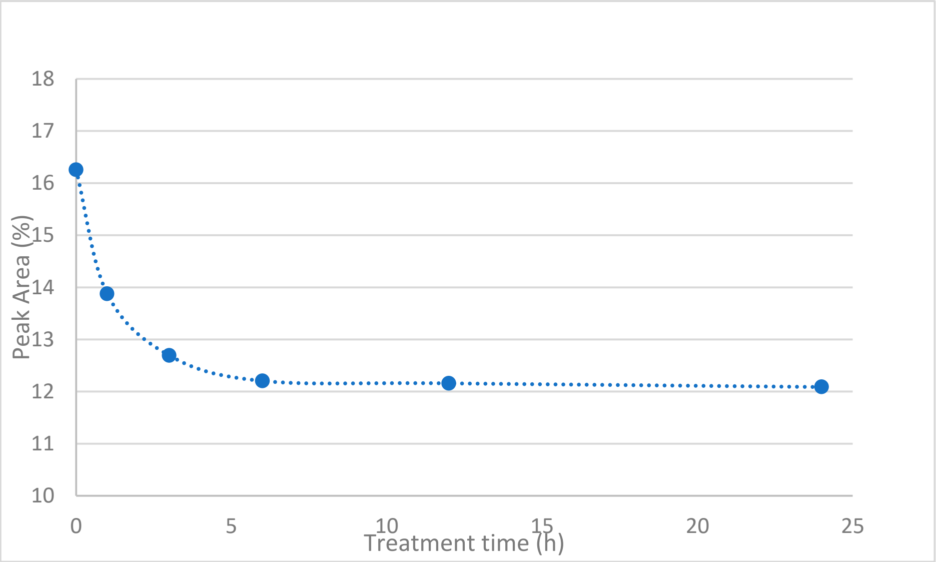

Figure S1. Relative area of the G band in the Raman spectra of acid-treated petcoke in percentage of total area in the 1000-1800 cm^{-1} region.

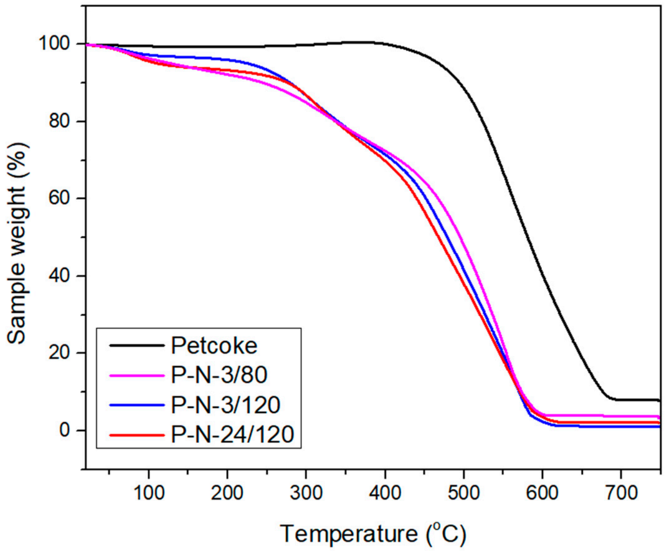

Figure S2. TGA profiles of petcoke and acid-treated petcoke (20 °C/min to 750 °C in a 50 mL/min flow of air).

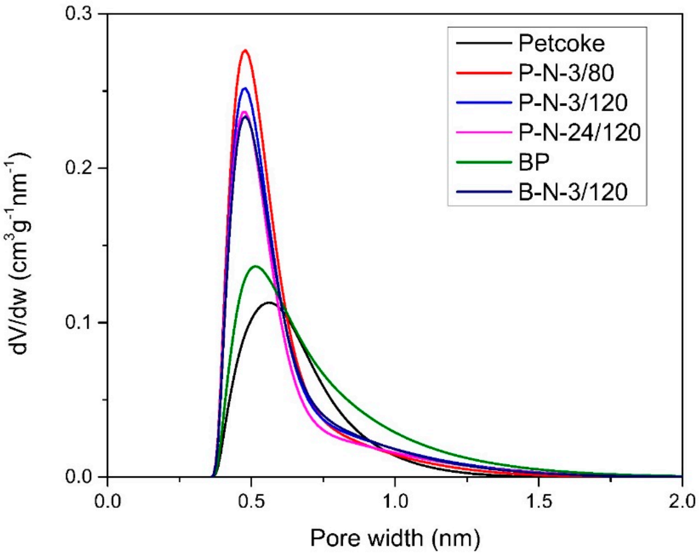

Figure S3. Pore size distributions of petcoke and petcoke-derived samples, as determined by CO_2 adsorption using a 2D-NLDFT-HS model.

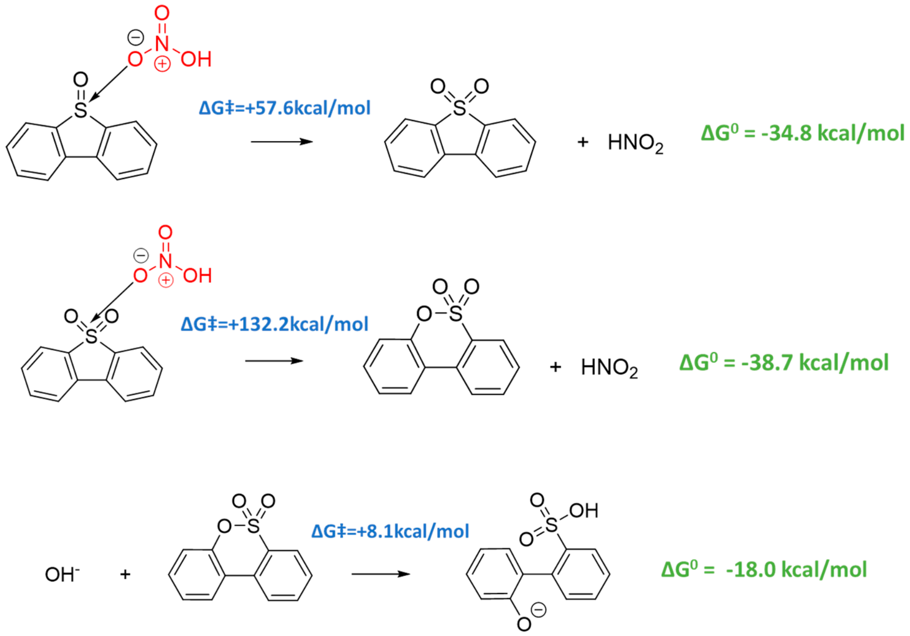

Figure S4. Reaction profile of DBT reacted with nitric acid (HNO_3).

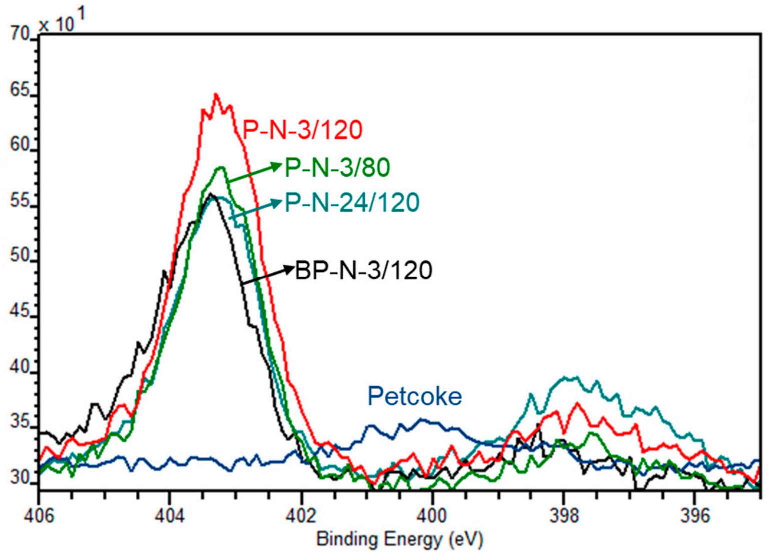

Figure S5. High-resolution XPS spectra of N 1s for petcoke derived solid acid catalysts.

N1s: before acid functionalization, there is little nitrogen on petcoke surface. The nitric acid resulted in the increase of nitrogen content and from the N1s spectra that -NO_2 groups are the dominated nitrogen-containing groups, which the binding energy is at 405.6 eV. The peaks centered at ~400 eV were reported as N-O, pyrrolic or pyridinic structures.[1] However, it could be a result of conversion of the -NO_2 into -NH_2 by exposing to X-ray irradiation during the XPS measurement.[2]

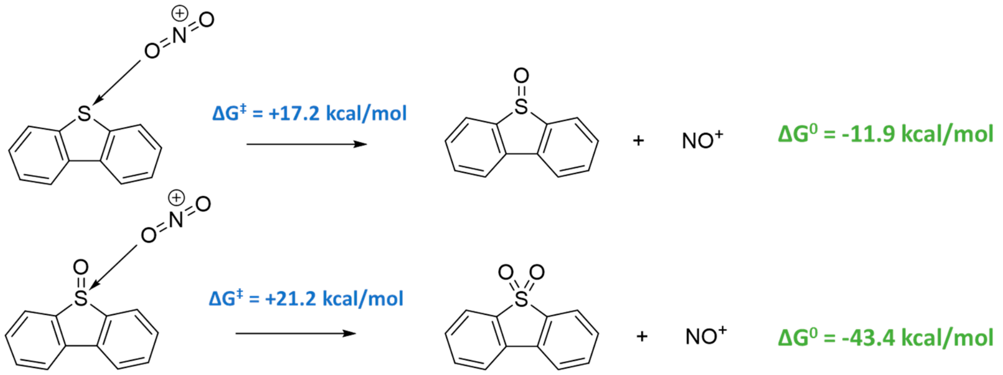

Figure S6. Reaction profile of DBT reacted with nitronium ion (NO_2^+).

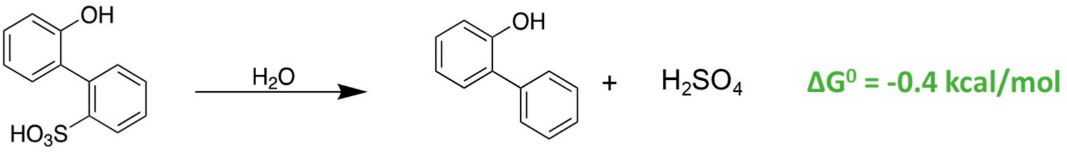

Figure S7. A spontaneous desulfurization reaction.

Table S1. Esterification of octanoic acid with methanol over different acid catalysts.

No.	Reactants	Catalyst (concentration of catalytic acid sites)	Reaction Conditions	Estimated TOF (h^{-1})	Productivity[3] ($g \cdot g^{-1} \cdot h^{-1}$)	Ref.
1	OA[1]+ MeOH[2]	H_2SO_4	60 °C, OA/MeOH = 1/40 (molar ratio)	87	52.5	3
2	OA+ MeOH	Silica Ball milled sulfonated petcoke (3.73 mmol/g)	60 °C, OA/MeOH = 1/40 (molar ratio)	85	10.3	3
3	OA+ MeOH	Ball milled sulfonated petcoke (1.58 mmol/g)	60 °C, OA/MeOH = 1/40 (molar ratio)	52	7.1	3
4	OA+ MeOH	Sulfonated petcoke (1.25 mmol/g)	60 °C, OA/MeOH = 1/40 (molar ratio)	48	5.6	3
5	OA+ MeOH	Sulfonated activated carbon from coffee residue (0.45-0.72 mmol/g)	60 °C, OA/MeOH = 1/3 (molar ratio)	25-47	4.0-6.0	4
6	OA+ MeOH	Amberlyst-15 (4.7 mmol/g)	60 °C, OA/MeOH = 1/3 (molar ratio)	4	3.9	4
7	OA + MeOH	Hollow sulfonated mesoporous carbon spheres (1.43 mmol/g)	75 °C, OA/MeOH =1/30 (molar ratio)	21.7	3.7	5
8	OA+ MeOH	Amberlyst-15 (4.7 mmol/g)	60 °C, OA/MeOH = 1/20 (molar ratio)	6	2.8	This work
9	OA+ MeOH	Ball milled nitric acid oxidized high sulfur petroleum coke (0.12 mmol/g)	60 °C, OA/MeOH = 1/20 (molar ratio)	143	1.9	This work
10	OA+ MeOH	Sulfonated chitosan (3.17 mmol/g)	60 °C, OA/MeOH = 1/95 (molar ratio)	114	1.3	6
11	Oleic acid +MeOH	Carbonized and sulfonated spent coffee grounds (3.36 mmol/g)	80 °C, oleic acid/MeOH = 1/10 (molar ratio)	-	1.3	7
12	OA+ MeOH	Nitric acid oxidized high sulfur petroleum coke (0.08 mmol/g)	60 °C, OA/MeOH = 1/20 (molar ratio)	114	1.1	This work
13	OA+ MeOH	Acid-activated clay (smectite) (0.57 mmol/g)	100 °C, OA/MeOH = 1/3 (molar ratio)	12	0.7	8

[1.] OA, octanoic acid; [2.] MeOH, methanol; [3.] gram of product per gram of catalyst and per hour.

References:

1. J.J. Ternero-Hidalgo, J.M. Rosas, J. Palomo, M.J. Valero-Romero, J. Rodríguez-Mirasol, T. Cordero, Functionalization of activated carbons by HNO3treatment: Influence of phosphorus surface groups, Carbon N. Y. 101 (2016) 409–419. https://doi.org/10.1016/j.carbon.2016.02.015.

2. P. Mendes, M. Belloni, M. Ashworth, C. Hardy, K. Nikitin, D. Fitzmaurice, K. Critchley, S. Evans, J. Preece, A novel example of X-ray-radiation-induced chemical reduction of an aromatic nitro-group-containing thin film on SiO2 to an aromatic amine film, ChemPhysChem. 4 (2003) 884–889. https://doi.org/10.1002/cphc.200300699.

3. Xiao, Y.; Hill, J. M. Solid Acid Catalysts Produced by Sulfonation of Petroleum Coke : Dominant Role of Aromatic Hydrogen. Chemosphere 2020, 248, 125981. https://doi.org/10.1016/j.chemosphere.2020.125981.

4. Ngaosuwan, K.; Goodwin, J. G.; Prasertdham, P. A Green Sulfonated Carbon-Based Catalyst Derived from Coffee Residue for Esterification. Renew. Energy 2016, 86, 262–269. https://doi.org/10.1016/j.renene.2015.08.010.

5. Lili Huang, Caicheng Song, Yingcen Liu, Hua Lin, Wanyue Ye, He Huang, Rongwen Lu, Shufen Zhang, Enhancement of catalytic esterification by tuning molecular diffusion in sulfonated carbon, Microporous and Mesoporous Materials, 2021, 318, 111024, https://doi.org/10.1016/j.micromeso.2021.111024 .

6. Castanheiro, J. Chitosan with Sulfonic Groups: A Catalyst for the Esterification of Caprylic Acid with Methanol. Polymers 2021, 13, 3924. https://doi.org/10.3390/polym13223924.

7. Agapay, R.C., Liu, HC., Ju, YH. et al. Synthesis and Initial Evaluation of Solid Acid Catalyst Derived from Spent Coffee Grounds for the Esterification of Oleic Acid and Methanol. Waste Biomass Valor, 2021, 12, 4387–4397. https://doi.org/10.1007/s12649-020-01325-6 .

8. Rezende, M. J. C.; Pinto, A. C. Esterification of Fatty Acids Using Acid-Activated Brazilian Smectite Natural Clay as a Catalyst. Renew. Energy 2016, 92, 171–177. https://doi.org/10.1016/j.renene.2016.02.004.
